# A Feasibility Study to Determine Whether Neuromuscular Adaptations to Equine Water Treadmill Exercise Can Be Detected Using Synchronous Surface Electromyography and Kinematic Data

**DOI:** 10.3390/ani15213189

**Published:** 2025-11-01

**Authors:** Lindsay St. George, Kathryn Nankervis, Victoria Walker, Christy Maddock, Amy Robinson, Jonathan Sinclair, Sarah Jane Hobbs

**Affiliations:** 1 Research Centre for Applied Sport, Physical Activity and Performance, University of Lancashire, Preston PR1 2HE, UK; jksinclair@lancashire.ac.uk (J.S.); sjhobbs1@lancashire.ac.uk (S.J.H.); 2 Equine Department, Hartpury University, Gloucester GL19 3BE, UK; kathryn.nankervis@hartpury.ac.uk (K.N.); victoria.walker@hartpury.ac.uk (V.W.); christy.maddock2@hartpury.ac.uk (C.M.); 3 Delsys Europe, Sale, Greater Manchester M33 2DH, UK; amy@delsyseurope.com

**Keywords:** equine, rehabilitation, biomechanics, hydrotherapy, muscle activity

## Abstract

**Simple Summary:**

Water treadmill (WT) exercise is increasingly used in equine training and rehabilitation programmes. Its use is supported by a growing body of research on the adaptive movements that horses adopt during both dry treadmill (TM) and WT exercise. Adaptive movement during WT exercise is presumed to be driven by underlying changes in muscle activity, but research has not yet confirmed this. This study aimed to evaluate the feasibility of a method for measuring synchronous movement and muscle activity patterns of horses executing WT exercise. Combined surface electromyography (sEMG) and three-dimensional (3D) kinematic technologies were used to collect hindlimb and trunk muscle activity, and back and pelvis movement data from one horse during overground (OG), TM, and WT walking conditions. These data were compared using statistical parametric mapping, which revealed distinct, significant differences (*p* > 0.05) in muscle activity and movement between conditions. Findings from this horse demonstrate that the proposed method is capable of quantifying equine movement and muscle activation patterns during WT exercise. Future applications of the research method described here will improve our understanding of equine muscle function during TM and WT exercise, allowing trainers and practitioners to design training and rehabilitation programmes that are backed by science.

**Abstract:**

Despite growing evidence on the adaptive movement patterns that horses adopt during water treadmill (WT) exercise, underlying adaptations in muscle activity remain uninvestigated. This feasibility study aimed to develop a method for the synchronous measurement of muscle activity and movement of horses during WT exercise. Combined surface electromyography (sEMG) (2000 Hz) from selected hindlimb (biceps femoris, gluteus medius, tensor fasciae latae) and epaxial (longissimus dorsi) muscles, and three-dimensional kinematic (200 Hz) data from the back and pelvis of one (n = 1) horse were collected during overground (OG), dry treadmill (TM), and WT walking conditions. Statistical parametric mapping evaluated differences in time- and amplitude-normalised sEMG and thoracolumbar and pelvis kinematic waveforms between conditions. Distinct, significant (*p* < 0.05) adaptations in hindlimb and epaxial muscle activation patterns and axial and pelvic kinematics, were observed in this horse across exercise conditions. Adaptive muscle activity was most pronounced in this horse during WT, compared to OG walking. These findings demonstrate the feasibility of this method, which combines sEMG and motion capture technologies to synchronously quantify equine movement and muscle activation patterns during WT exercise. This justifies the replication of this work in a larger sample of horses to inform evidence-based training and rehabilitation programmes.

## 1. Introduction

When training and/or recovering horses from injury, the opportunity to develop targeted programmes specific to the individual’s needs is more challenging than in human athletes due to the absence of patient cognisance and motivation. Exercise or rehabilitation programmes for horses are based on clinical reasoning that supports the desired movement patterns and/or targeted recruitment of specific muscle groups [1]. Thus, developing a better understanding of the physiological and biomechanical responses invoked by the available exercise modalities is a prerequisite for employing an evidence-based approach to equine training and rehabilitation. Of these, dry treadmill (TM) and water treadmill (WT) walking are commonly employed and represent low-intensity aerobic exercise [2], which is advocated in the early stages of training [3] and rehabilitation [4,5,6] of horses. The movement patterns that horses adopt during these forms of exercise have been quantified [7,8,9,10,11,12,13,14] and are assumed to be perpetuated by adaptive muscle activation [8,11,15]. However, no studies have confirmed this assumption for WT exercise through the combined evaluation of muscle activation and the resultant movement patterns. Thus, to develop the evidence base for the use of WT within equine training and rehabilitation programmes, it is prudent to develop research methods to synchronously quantify the movement and underlying neuromuscular control patterns of horses executing this form of exercise.

During rehabilitation, the horse’s return to exercise often starts with hand walking overground (OG) [16]. Treadmill exercise can be used as an alternative to OG hand walking as a method of controlled, non-ridden, and straight-line exercise [5]. However, the known differences in limb and back kinematics that occur between OG and TM walking [7,17,18] must be considered when developing rehabilitation plans for horses. For example, the treadmill belt elicits passive retraction during the stance phase, which significantly increases fore- and hindlimb retraction during TM compared to OG walking [7]. As the horse progresses through a rehabilitation or training programme, targeted increases in muscle activation may be induced on a treadmill through modifications in speed [19,20], incline [21], and/or the addition of water [22,23]. To date, the effects of WT exercise on equine limb [8,9,10,11,15] and back kinematics [10,12,13,14] have been described. As such, WT exercise is often incorporated into the training and rehabilitation programmes of sport horses and racehorses [24,25,26], with physical properties of water such as buoyancy and hydrostatic pressure reported to improve rehabilitation outcomes [26,27,28,29,30]. To date, however, the kinematic changes have not yet been directly related to associated changes in muscle activity.

When moving through water, changes in movement patterns are observed when compared to OG or TM locomotion. During the swing phase, the limb is assisted when moving towards the water surface due to buoyancy but is opposed when moving directly forward due to the viscosity of the water [15,22,29]. In water depths below the carpus and hock joints, which are commonly used for training and rehabilitation purposes [24], horses tend to exhibit greater elevation and flexion of the distal hindlimb joints than when moving on a dry belt [8]. The effect of assisting fluid forces can further increase hindlimb retraction during WT compared to TM exercise [11]. Whilst the net effect of fluid forces has a modest impact on the workload of the horse during TM and WT, as measured using oxygen uptake [23], the impact on work performed by individual muscles may be significant during certain phases of the stride. In support of this, Tokuriki et al. [22] found that activation of selected forelimb and neck muscles was up to three times greater during WT compared to OG walking. Emerging evidence also supports the repeated use of WT exercise to increase hindlimb muscle development in horses as measured both quantitatively using ultrasound [31,32] and using visual, subjective assessment [33]. Taken together, the known WT-induced changes in hindlimb movement and muscle morphology are assumed to be perpetuated by adaptive muscle activation patterns but have yet to be quantified in horses. The lack of hard evidence represents a significant gap in knowledge, as equine training and rehabilitation programmes should be evidence-based for optimised efficacy and management of injury risk.

In recognition of this, a feasibility study was conducted with the aim of developing a method for the synchronous measurement of muscle activity and movement of horses during WT exercise. To fulfil this aim, we collected combined surface electromyography (sEMG) data from selected hindlimb and axial muscles and three-dimensional (3D) kinematic data from the back and pelvis of one horse during OG, TM, and WT walking conditions. We hypothesised that a comparison of continuous time-series data between OG and treadmill (TM and WT) conditions would reveal that combined sEMG and kinematic data are capable of detecting adaptations in muscle activity and movement across the walk stride cycle.

## 2. Materials and Methods

One (n = 1) privately owned, healthy adult horse (age: 10 years, height: 155 cm, breed: Belgian Warmblood, sex: mare) participated in this feasibility study. The horse was housed at Hartpury University, where the study was conducted, was in consistent work of 6 sessions/week (hack × 2, TM or WT × 1 and schooling × 3) and competed in showjumping at 90–100 cm once or twice a month. The horse was fully acclimated to WT exercise [34] on the model (Aqua Equine, Carlow, Ireland) used for the study. Ethical approval for this study was obtained from the Hartpury University Ethics Committee (Ref: ETHICS2023-114). Written informed consent was obtained from the horse owner prior to the study.

### 2.1. Horse Preparation

Muscle activation and 3D kinematic data were collected from the horse using combined sEMG and inertial measurement unit (IMU) sensors (Trigno Avanti, Delsys Inc., Natick, MA, USA) and an optical motion capture (OMC) system (Qualisys AB, Göteborg, Sweden). Retro-reflective markers were attached over the following anatomical landmarks using double-sided adhesive tape to acquire OMC data from the axial skeleton and pelvis: approximately over the T5, L1, and S3 vertebrae, between the tubera sacrale (TS), and the proximal aspect of the left and right tuber coxae (Supplementary Figure S1). Markers were also attached to each corner of the water treadmill for reference. In addition, IMU sensors (Trigno Avanti, Delsys Inc., Natick, MA, USA) were placed over the poll, withers (approximately over T5 vertebrae) and between the TS to acquire upper-body movement data (Supplementary Figure S1). Retro-reflective markers (25 mm diameter) were situated directly on top of the IMU sensors attached over the withers and between the TS.

sEMG sensors (Trigno Avanti, Delsys Inc., Natick, MA, USA) were positioned to acquire muscle activity data from selected superficial muscles of the back and hindlimb that were purported to exhibit adaptive activation patterns, based on the reported changes in associated kinematics that occur in relation to the demands of WT exercise [10,11,13]. Specifically, sEMG sensors were unilaterally positioned to record from the horse’s right gluteus medius (GM), biceps femoris (BF), tensor fasciae latae (TFL), and bilateral longissimus dorsi (LD) (Supplementary Figure S1). Sensor sites for each muscle were as follows: LD: at the L1 vertebrae, 6 cm lateral to midline [35,36]; GM: cranial to the greater trochanter at a point approximately midway between the tubera sacrale and greater trochanter [37]; BF: approximately midway between the greater trochanter and patella, 12–18 cm cephalad to the cranial margin of semitendinosus [38,39]; TFL: approximately one quarter of the distance between the ventral tuber coxae and the lateral epicondyle of the femur [40,41]. Each sensor site was prepared by first removing hair using clippers and then thoroughly cleaning the skin using isopropyl alcohol. A small amount of saline solution was applied to the electrode bars to act as an electrolytic solution. Sensors were then adhered to prepared skin using a combination of Delsys Adhesive Surface Interface Strips (Delsys Inc., Natick, MA, USA) and strips of double-sided tape. Sensors were positioned on the muscle belly, with the electrodes orientated perpendicular to the underlying muscle fibre direction [42,43]. For the WT condition, protective pouches were constructed for the sEMG sensors on the BF and TFL locations to catch the sensor should it become detached. Strips of water-resistant adhesive tape were used to create the pouches, which adhered to the horse at four points and had a raised, non-adhesive, non-contact section in the middle for the sensor to sit beneath. The sensors and markers remained in place across all conditions to enable direct comparisons.

### 2.2. Data Acquisition Protocol

Four Qualisys Miqus cameras (Qualisys AB, Göteborg, Sweden) were positioned 1.5 m behind the water treadmill at a height of 3 m and calibrated to produce a capture volume for acquiring 3D kinematic data from the axial and pelvic markers. As the OG trials took place outside of the treadmill room and only one OMC system was available for this preliminary study, it was not feasible to collect OMC data for the OG condition.

Optical motion capture (200 Hz), IMU (2000 Hz), and sEMG (2000 Hz) data were collected synchronously using an external trigger system (Delsys Trigger Module, Delsys Inc., Natick, MA, USA) and Qualisys Track Manager (version 2023.2, Qualisys AB, Göteborg, Sweden) software. Horses generally execute WT walking exercise at approximately 80% of overground speed [44], so dry treadmill conditions (i.e., water treadmill without water) were speed matched to both overground (TM) and WT (TM_80_) conditions to enable direct comparisons across conditions. Data were collected in walk across four conditions: OG, water treadmill (WT_80_), and dry treadmill (TM and TM_80_). The horse wore its normal bridle across all conditions. Initially, sEMG and IMU data were synchronously collected during the OG condition, which was conducted outdoors on a flat, asphalt track that was approximately 30 m in length. Following a 5-min warm-up of OG walking, four in-hand walking trials were conducted for the OG condition, with the horse permitted to walk at its preferred speed. From here, OMC, sEMG and IMU data were synchronously collected from a static trial, with the horse standing square on the dry treadmill, followed by treadmill conditions, executed at walk in the following order: TM (1.7 m/s), TM_80_ (1.4 m/s), and WT_80_ (1.4 m/s, water depth: mid-metatarsal). Mid-metatarsal water depth was used for the WT_80_ condition, as it is commonly used in training and rehabilitation practice [24]. There was an approximately 5 min break in between OG and treadmill data collection. Data were collected for 60 s from two trials across treadmill conditions (TM, TM_80_, and WT_80_), following a 5 min warm-up under the TM condition.

### 2.3. Data Analysis

Kinematic data were tracked in Qualisys Track Manager (version 2023.2, Qualisys AB, Göteborg, Sweden), and data files were imported into Visual3D (version 2021.06.2, HAS-Motion Inc., Kingston, ON, Canada) software for post-processing.

#### 2.3.1. Kinematic Data

Stride segmentation was based on the detection of right hindlimb impact events, defined as the vertical displacement minima of the TS, in accordance with the method described by Roepstorff et al. [45]. Vertical displacement of the TS was derived from IMU data, as these were available across both OG and treadmill conditions. Vertical acceleration data from the IMU placed between the TS was integrated twice, removing the mean from the signal following each integration step [46], to obtain vertical displacement signals. These signals were high-pass filtered (Butterworth 4^th^-order) with a cut-off frequency that was adjusted to the stride frequency of the horse [47] and then low-pass filtered (Butterworth 4^th^-order, 30 Hz cut-off) [48]. Kinematic gait events were applied to sEMG and kinematic signals from each trial for stride segmentation and analysis.

A rigid-body segment model of the axial skeleton and pelvis was created using OMC data from the treadmill conditions (Supplementary Figure S2), in accordance with the method described by Hobbs et al. [49]. A thoracolumbar angle was calculated using a cranial segment, defined using markers located on the T5 and L1 vertebrae, and a caudal segment, defined using markers on the L1 vertebra and the TS. Thoracolumbar flexion/extension and lateral bending angles were defined in the sagittal and transverse planes, respectively, where flexion and bending to the right were defined as positive and extension and bending to the left as negative. A pelvic segment was created using the left and right tuber coxae as proximal end markers and the anatomical S3 marker and a virtual marker, created 2 cm to the left side of the S3 marker [49], as distal end markers. Pelvic angles were calculated relative to a reference body segment, defined using the T5 vertebral and TS markers. Pelvis pitch was defined as positive during flexion and negative for extension, and pelvis roll and yaw rotations were, respectively, defined as positive for downward (ventral) and forward (cranial) movements of the right tuber coxae relative to the left tuber coxae and vice versa for negative rotations. Thoracolumbar and pelvis angles were calculated using the Cardan sequence x, y, z and were low-pass filtered using a Butterworth 4^th^-order filter with a 30 Hz cut-off frequency [36,50]. Sagittal plane thoracolumbar and pelvic angles were normalised to the corresponding static angles to differentiate between flexion and extension. Thoracolumbar lateral bending and pelvis roll and yaw angles were not normalised, as their calculation is based on an anatomically neutral position and thus differentiated through a 0-degree crossing.

#### 2.3.2. sEMG Data

Raw sEMG signals were DC-offset removed, high-pass filtered using a Butterworth 4th-order filter with a 40 Hz cut-off frequency [51], and full-wave rectified. Rectified signals were smoothed using a Butterworth 4^th^-order, low-pass filter with a 10 Hz cut-off frequency, which is based on previous studies evaluating muscle activity in horses at walk on a treadmill [52,53,54]. The peak amplitude (PA) value was extracted using low-pass filtered signals from each muscle across strides and conditions. PA was used to detect and remove outlier strides according to the method described by St George et al. [38]. From here, low-pass filtered signals were normalised to a reference voluntary contraction (RVC), defined as the maximum PA value observed across OG strides within each muscle. This normalisation technique permitted examination of the proportional difference in muscle activity between OG and treadmill walking conditions.

### 2.4. Statistical Analysis

One-dimensional statistical parametric mapping (SPM) was used to analyse differences between OG and treadmill conditions (TM, TM_80_, WT_80_) using continuous, time-series data from amplitude-normalised sEMG data. SPM was also used to analyse differences in thoracolumbar and pelvis kinematics across treadmill conditions, as these data were not available from the OG condition. For comparative purposes, the same comparisons across treadmill conditions were conducted for sEMG data using SPM. The time- and amplitude-normalised stride values for sEMG data and the angle-time curves for kinematic data were assembled into 1∗101∗1 vector fields (101 data points per stride and 1 dimension per data point) for each signal and condition. Importantly, as our sample consisted of one subject, intra-subject (stride-to-stride) variance was used to analyse sEMG and kinematic data using SPM. The analysis of time-series data from individual horses using SPM has been reported and endorsed in other equine biomechanics studies [36,37,55,56] and was thus considered suitable for this feasibility study. The same number of strides was analysed per condition by removing strides at the beginning and end of each trial following outlier removal. SPM was undertaken within MATLAB R2024a (v. 24.2, MATLAB, MathWorks, Natick, MA, USA), using source code available at http://www.spm1d.org/ (accessed on 16 April 2023). Significance was set at *p* < 0.05.

## 3. Results and Discussion

**Figure 1:**
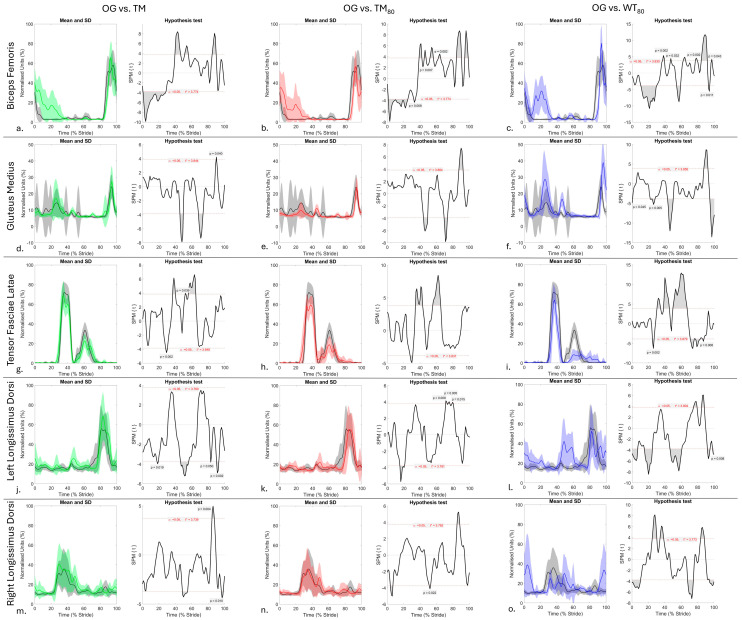
SPM results for time- and amplitude-normalised sEMG data from n = 1 horse for right biceps femoris (**a**–**c**), gluteus medius (**d**–**f**), tensor fasciae latae (**g**–**i**), and bilateral longissimus dorsi (**j**–**o**) between OG (black) and TM (green), TM_80_ (red), and WT_80_ (blue) walking conditions. Within each sub-panel, left-side graphs illustrate mean (solid line) and standard deviation (shaded area) sEMG data from individual muscles that are time normalised to walk stride duration. Right-side graphs illustrate paired samples *t*-test SPM results (black solid line) and the critical thresholds (α, t*) for significance (red dashed line), with grey shaded areas indicating regions/data clusters with statistically significant differences (*p* < 0.05) between conditions. *P*-values are presented for data clusters with significance between 0.001 and 0.05; all other shaded data clusters are *p* < 0.001.

**Figure 2:**
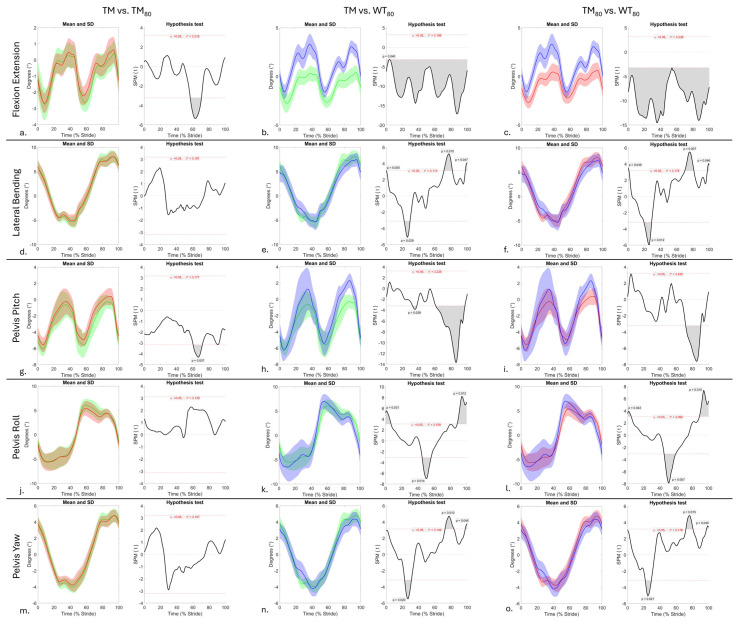
SPM results for time-normalised thoracolumbar flexion/extension (**a**–**c**) and lateral bending (**d**–**f**) angles, and pelvis roll (**g**–**i**), pitch (**j**–**l**), and yaw (**m**–**o**) angles (degrees) from n = 1 horse between treadmill walking conditions: TM (green), TM_80_ (red), and WT_80_ (blue). Within each sub-panel, left-side graphs illustrate mean (solid line) and standard deviation (shaded area) kinematic data that are time normalised to walk stride duration. The right-side graphs illustrate paired samples *t*-test SPM results (black solid line) and the critical thresholds (α, t*) for significance (red dashed line), with grey shaded areas indicating regions/data clusters with statistically significant differences (*p* < 0.05) between conditions. *P*-values are presented for data clusters with significance between 0.001 and 0.05; all other shaded data clusters are *p* < 0.001.

A total of 128 and 96 walk strides were included in the analysis of sEMG (4 conditions, 32 strides per condition) and kinematic (3 conditions, 32 strides per condition) data, respectively, using SPM. SPM results for sEMG data are presented in Figure 1 and revealed significant differences (*p* < 0.05) in activation across all muscles between OG and treadmill (TM, TM_80,_ and WT_80_) conditions, which are described and discussed in the following sections. SPM results for the comparison of kinematic data across treadmill conditions are presented in Figure 2 and are integrated into the discussion of sEMG results. Supplementary Figures provide an illustrative comparison of mean ± standard deviation (SD) sEMG signals from each muscle (Figure S3) and thoracolumbar and pelvic angles (Figure S4) across conditions, as well as SPM results for the comparison of sEMG data across treadmill conditions (Figure S5).

### 3.1. Hindlimb Muscle Activity

During OG walk, this horse exhibited BF activity from late swing phase to early stance phase (Figure 1a–c and Figure S3a), with the TFL exhibiting a clear biphasic activation pattern that was characterised by an initial, higher amplitude burst from mid- to late stance phase and a second, lower amplitude burst during the first half of swing phase (Figure 1g–i and Figure S3c). These activation patterns for BF and TFL agree with previous electromyographic studies of horses during OG walking [57,58,59]. In this horse, the observed activation pattern for GM agrees with other electromyographic studies of OG walking that reported activation from late swing phase to approximately mid-late stance phase [57,58]. Although studies have employed sEMG to quantify hindlimb muscle activation under dry TM conditions, these have largely been conducted at trot [19,20,40], with limited studies evaluating walk [21,60]. This represents a gap in knowledge considering the frequent use of walking for training and rehabilitation purposes [5,16]. To our knowledge, this feasibility study is the first to quantify BF, GM and TFL activation during TM and WT walking conditions.

#### 3.1.1. Biceps Femoris and Gluteus Medius

During TM, TM_80_, and WT_80_ conditions, the GM exhibited a clear biphasic activation pattern in stance, with the first burst occurring at mid-stance (10–40% stride), followed by a comparatively shorter, lower-amplitude burst at late stance (40–50% stride) (Figure 1d–f). Clear and significant increases in BF activity were also observed at mid-stance between 10 and 40% of stride duration across TM, TM_80_ and WT_80_ conditions when compared to OG (*p* < 0.001) (Figure 1a–c), but significant increases in GM activity at mid-stance were only observed during WT_80_ (*p* < 0.001) (Figure 1f). In contrast, the second burst of GM activation during late stance (40–50% stride) resulted in significant increases (*p* < 0.001) in muscle activation across TM, TM_80_, and WT_80_ conditions when compared to OG (Figure 1d–f). Although limb kinematics were not measured here, which represents a limitation of this work, previous studies have demonstrated significant increases in hindlimb retraction during dry TM exercise [7] that are exacerbated by increases in belt speed and water depth [10,11,13]. No known studies have quantified hip or stifle joint ROM during TM or WT walking, but authors have hypothesised that increased hip extension occurs during WT exercise due to belt movement and water resistance [11]. Indeed, the increased and prolonged co-activation of the hip extensors studied here (BF and GM) at mid-stance during TM, TM_80_, and WT_80_ conditions would support this hypothesis and may reflect a requirement for greater muscular contribution to stabilise the hindlimb as it undergoes loading and both active and passive retraction during treadmill exercise. This is further supported by our finding that increases in BF and GM activation were most prominent in this horse during the WT_80_ condition, where increases in hip and stifle extension were visually observed at mid-stance, as the contralateral hindlimb underwent swing phase. This suggests that water treadmill locomotion requires increased recruitment of BF and GM to stabilise the joints that they work on.

During WT_80_, we observed that this horse adopted the strategy described by Mendez-Angulo et al. [8] by flexing the hindlimb joints to increase flight arc and partially clear the water surface during swing phase. The duration of swing phase is significantly prolonged during WT exercise [8], which may explain the significant delay (*p* < 0.001) in BF and GM activation that was observed here in late swing phase during WT_80_ (Figure 1c, Figure 1f, respectively) when compared to OG. However, further research is required to understand why significant delays in BF and GM activation (*p* < 0.001) were also observed during TM and TM_80_ conditions (Figure 1a,b,d,e), albeit to a lesser degree than WT_80_. When compared to OG, significant increases in amplitude were also observed during late swing phase in both BF and GM across conditions (*p* < 0.05), except for GM during TM_80_, which did not differ significantly from OG (Figure 1e). These increases in GM and BF activity were also most pronounced in this horse during WT_80_ and coincide with reversal of the limb’s direction of movement for impact. As such, these significant increases in sEMG amplitude may represent greater active contributions of BF and GM to facilitate extension of the hip and stifle joints to initiate retraction and possibly to break through water surface tension prior to impact. Our interpretation of these findings from a single horse should be considered with caution, and further research is required to confirm whether similar muscle activation patterns are observed in a larger sample of horses. This is especially important, as studies have reported inter-horse variation in limb movement strategies during WT exercise at differing water depths [8,10,11], which we postulate are facilitated by different neuromuscular strategies.

#### 3.1.2. Tensor Fasciae Latae

In contrast to BF and GM, significant decreases (*p* < 0.001) in the peak amplitude of TFL activation bursts during late stance and early swing phases were observed during TM, TM_80_ and WT_80_ conditions when compared to OG (Figure 1g–i). In this horse, alterations in the phasic activation pattern of TFL were most pronounced during WT_80_ when compared to OG, with significantly shorter and prolonged activation bursts during stance and swing phase (*p* < 0.001), respectively, and with significantly decreased amplitude (*p* < 0.001) across the WT_80_ stride cycle (Figure 1i). During WT exercise, the treadmill belt and buoyancy assist the limb in moving towards the water surface [15], which may reduce the need for active muscular contributions from the TFL during stance to flex the hip joint and stabilise the flexed stifle. Interestingly, in comparison to OG, TFL activation was significantly longer in swing phase during WT_80_ (*p* < 0.01, Figure 1i), remaining active until BF and GM activation. It is possible that the TFL remains active for longer during WT exercise to maintain prolonged increases in hip and stifle joint flexion to clear the water surface during swing phase. It is also possible that TFL activation is decreased across the stride cycle because treadmill exercise elicits increased activation of other hip flexors, like the quadriceps, which exhibits increased activation during dry [61,62,63] and water treadmill [64] locomotion in humans when compared to OG walking. In this horse, co-activation of BF and GM was observed during late stance phase in treadmill conditions, but not during OG walking, which could also mitigate active contributions from the TFL, as was observed by Eldridge et al. [58] during backward walking in horses. Again, further research is required to better understand these adaptive activation patterns in TFL using combined sEMG and kinematic data from the hindlimbs.

To summarise, the adaptations in GM, BF, and TFL activity were most prominent in this horse during WT_80_ when compared to OG and were generally not observed between TM and TM_80_ conditions (*p* > 0.05) (Supplementary Figure S5a,d,g), suggesting that muscle activation may be more affected by the presence of water than treadmill speed, which agrees with kinematic studies [13,65]. However, further work is required to confirm this using sEMG in a larger group of horses. Importantly, our findings from this horse provide quantitative support for the postulation put forth by Nankervis et al. [5] that WT exercise requires muscle activation patterns that differ from walking OG and on a dry treadmill. The general increases in GM and BF activation observed in this horse during WT_80_ also appear to support Mendez-Angulo et al. [8] and Nankervis and Lefrancois [11], who advise careful consideration for muscles that act to pro-retract the limb when developing WT programmes, as these may be more prone to fatigue or injury in horses naïve to this type of exercise. Further research on a larger sample of horses is required to confirm whether adaptations in muscle activation patterns support the use of WT exercise for the development of hindlimb strength in horses.

### 3.2. Longissimus Dorsi and Axial Kinematics

Interindividual variation in the phasic activation pattern of LD has been described during walking and trotting [36,66], which highlights the cautious approach that must be taken when interpreting results from the single horse studied here. During OG, TM, and TM_80_ conditions, this horse exhibited a monophasic burst of longissimus lumborum activity between the mid- and late stance phases of the ipsilateral hindlimb (Figure 1j,k,m,n), which agrees with other studies [52,67] and coincides with the initiation of lateral bending to the ipsilateral side and thoracolumbar flexion (Figure 2a,d). The lack of kinematic data from the OG condition is a limitation of this study, and to our knowledge, no studies have compared axial kinematics between OG and treadmill walking conditions. This makes interpretation of differences in LD activity between OG and treadmill conditions difficult. In this horse, bilateral LD activity was similar between OG, TM, and TM_80_ conditions (Figure 1j,k,m,n and Figure S5j,m). The comparable axial movement patterns observed here between TM and TM_80_ conditions (Figure 2a,d,g,j,m), and between OG and dry TM conditions in the trotting horse [17] could explain the general similarities in LD activity between OG, TM, and TM_80_ walking conditions. Further research is required to confirm this. Additionally, the general trend for non-significant differences in LD activity between TM and TM_80_ conditions (Supplementary Figure S5j,m) agrees with Licka et al. [52], who found that dry treadmill walking speed was not significantly correlated with the amplitude and timing of the LD sEMG maxima. Again, further research is required to confirm our interpretation of these findings in a larger sample of horses.

In contrast to TM and TM_80_ conditions, WT_80_ elicited significant adaptations in the bilateral phasic activity pattern of this horse’s LD when compared to OG. These adaptations were characterised by a biphasic LD activation pattern, resulting in bilateral co-activation during late stance phase and early-swing phase of each hindlimb and corresponding to peak thoracolumbar and pelvic flexion and lateral bending (Figure 2b,c,e,f,h,i and Figure S4a–c). This phasic activation pattern agrees with that described by Wakeling et al. [35] during dry TM walking conditions at comparable speed (1.4 m/s) on both a 0° and 10° incline. Previous equine sEMG studies have demonstrated that significant increases in LD activation occur during faster gaits [68] and on an incline [69]. This is expected to be a means of stabilising the more rigid axial skeleton, especially in comparison to level walking [69,70,71]. Our findings suggest that increased LD activation also occurred in this horse to adapt to the demands of WT exercise when compared to the OG condition. However, our lack of kinematic data from the OG condition and the fact that no other studies have directly compared axial movement or muscle activity between OG and WT conditions limit our ability to interpret the adaptations in LD activation that were observed here. Thus, further research is required to better understand differences in axial muscle activity and resultant movement between WT and OG walking.

Despite this, a comparison of LD activation and axial kinematics between dry treadmill and WT_80_ conditions offers a preliminary insight into the adaptations that are elicited by WT exercise (Figure 2 and Figure S5). During WT_80_, the main burst of LD activity exhibited significantly longer activation and decreased amplitude when compared to TM and TM_80_ conditions (*p* < 0.05, Supplementary Figure S5k,l,n,o). The second burst of LD activity, observed during the WT_80_ condition, led to significant increases in sEMG amplitude during early stance and late swing phases of the ipsilateral hindlimb when compared to the TM and TM_80_ conditions (*p* < 0.01, Supplementary Figure S5k,l,n,o). These adaptations coincided with a significant increase and decrease in peak thoracolumbar flexion and extension (*p* < 0.001), respectively, as well as a significantly prolonged period of flexion and shortened period of extension (*p* < 0.001) during WT_80_ compared to TM and TM_80_ conditions (Figure 2b,c). The consistent upward displacement of the flexion/extension curve is hypothesised as being related to the horse adopting a “nose down” posture where increased vertical displacement of the croup and decreased displacement of the withers were visually observed and confirmed using OMC data. A similar trend was observed in this horse’s pelvis pitch and roll movement during WT_80_ (Figure 2h,i,k,l), where significant increases in peak flexion (*p* < 0.001) and roll (*p* < 0.05) occurred during WT_80,_ except for peak flexion during right hindlimb stance phase (*p* > 0.05). These general increases in thoracolumbar flexion and pelvic flexion and roll, without increases in thoracolumbar lateral bending (Figure 2e,f,n,o), agree with other studies that describe the same kinematic changes at comparable water depth and speed [10,13]. Nankervis et al. [13] proposed that non-significant differences in thoracolumbar lateral bending between dry and WT exercise are due to increases in bilateral paraspinal muscle activity for spinal stability. Our findings support this notion and suggest that this horse increased longissimus lumborum activation during WT_80_ to stabilise the spine against increases in thoracolumbar and pelvic ROM, which are thought to be passively driven by changes in hindlimb movement [10,13,72]. Further studies are required to understand the relationship between axial movement and activity at different regions of the longissimus dorsi muscle [35] that occur during WT exercise.

## 4. Conclusions

Findings from this case study demonstrate that dry and water treadmill exercise elicited significant adaptations in hindlimb and epaxial muscle activation patterns when compared to OG walking. In addition, findings demonstrate that dry and water treadmill exercise elicit differing adaptations in axial and pelvic kinematics. Importantly, our study demonstrates the feasibility of using combined sEMG and motion capture technologies to synchronously quantify equine movement and muscle activation patterns during water treadmill exercise. Our use of SPM to evaluate differences in continuous sEMG and 3D kinematic time-series data revealed distinct, significant adaptations to each exercise condition across the stride cycle. Although findings from this case study should be interpreted with caution given the sample size of n = 1, they justify and demonstrate the importance of replicating this work in a larger sample of horses. Future applications of the methods described here will improve our understanding of the activity of major locomotor muscles during dry and water treadmill exercise with direct comparison to OG exercise, which will inform evidence-based decision-making for trainers and clinicians alike.

## Data Availability

The raw data supporting the conclusions of this article will be made available by the authors on request.

## Supplementary Materials

The following supporting information can be downloaded at: https://www.mdpi.com/article/10.3390/ani15213189/s1, Figure S1: Retro-reflective markers and surface electromyography (sEMG) sensors attached to the horse.; Figure S2: Rigid-body segment model and derived kinematic angles for (a) thoracolumbar flexion/extension, (b) thoracolumbar lateral bending, (c) pelvis pitch, (d) pelvis yaw, (e) pelvis roll.; Figure S3: Mean and standard deviation time- and amplitude-normalised sEMG signals from (a) biceps femoris, (b) gluteus medius, (c) tensor fasciae latae, (d) left longissimus dorsi, (e) right longissimus dorsi across OG, TM, TM_80_, and WT_80_ walking conditions.; Figure S4: Mean and standard deviation time-angle curves for (a) thoracolumbar flexion/extension, (b) thoracolumbar lateral bending, (c) pelvic pitch, (d) pelvic roll, (e) pelvic yaw across TM, TM_80_, and WT_80_ walking conditions; Figure S5: SPM results for time- and amplitude-normalised sEMG data from n = 1 horse for right hindlimb muscles and bilateral longissimus dorsi between treadmill walking conditions.

## Abbreviations

The following abbreviations are used in this manuscript:

3D	Three-Dimensional
BF	Biceps Femoris
GM	Gluteus Medius
IMU	Inertial Measurement Unit
LD	Longissimus Dorsi
OG	Overground
OMC	Optical Motion Capture System
PA	Peak Amplitude
RVC	Reference Voluntary Contraction
sEMG	Surface Electromyography
TFL	Tensor Fasciae Latae
TM	Treadmill
TS	Tubera Sacrale
WT	Water Treadmill

## References

[1] Tabor G., Williams J. (2018). Equine rehabilitation: A review of trunk and hind limb muscle activity and exercise selection. J. Equine Vet. Sci..

[2] Voss B., Mohr E., Krzywanek H. (2002). Effects of aqua-treadmill exercise on selected blood parameters and on heart-rate variability of horses. J. Vet. Med. Ser. A.

[3] Clayton H.M. (1991). Conditioning Sport Horses.

[4] Haussler K., King M., Peck K., Adair H. (2021). The development of safe and effective rehabilitation protocols for horses. Equine Vet. Educ..

[5] Nankervis K.J., Launder E.J., Murray R.C. (2017). The use of treadmills within the rehabilitation of horses. J. Equine Vet. Sci..

[6] Goff L., Stubbs N. (2007). Equine treatment and rehabilitation. Anim. Physiother..

[7] Buchner H., Savelberg H., Schamhardt H., Merkens H., Barneveld A. (1994). Kinematics of treadmill versus overground locomotion in horses. Vet. Q..

[8] Mendez-Angulo J.L., Firshman A.M., Groschen D.M., Kieffer P.J., Trumble T.N. (2013). Effect of water depth on amount of flexion and extension of joints of the distal aspects of the limbs in healthy horses walking on an underwater treadmill. Am. J. Vet. Res..

[9] McCrae P., Bradley M., Rolian C., Léguillette R. (2021). Water height modifies forelimb kinematics of horses during water treadmill exercise. Comp. Exerc. Physiol..

[10] Tranquille C., Tacey J., Walker V., Mackechnie-Guire R., Ellis J., Nankervis K., Newton R., Murray R. (2022). Effect of Water Depth on Limb and Back Kinematics in Horses Walking on a Water Treadmill. J. Equine Vet. Sci..

[11] Nankervis K.J., Lefrancois K. (2018). A comparison of protraction-retraction of the distal limb during treadmill and water treadmill walking in horses. J. Equine Vet. Sci..

[12] Mooij M., Jans W., Den Heijer G., De Pater M., Back W. (2013). Biomechanical responses of the back of riding horses to water treadmill exercise. Vet. J..

[13] Nankervis K., Tranquille C., Chojnacka K., Tacey J., Deckers I., Newton J., Murray R. (2023). Effect of speed and water depth on limb and back kinematics in Thoroughbred horses walking on a water treadmill. Vet. J..

[14] Nankervis K., Finney P., Launder L. (2016). Water depth modifies back kinematics of horses during water treadmill exercise. Equine Vet. J..

[15] Scott R., Nankervis K., Stringer C., Westcott K., Marlin D. (2010). The effect of water height on stride frequency, stride length and heart rate during water treadmill exercise. Equine Vet. J..

[16] Goff L. (2016). Equine sports medicine and performance management. Animal Physiotherapy: Assessment, Treatment and Rehabilitation of Animals.

[17] Alvarez C.G., Rhodin M., Byström A., Back W., Van Weeren P. (2009). Back kinematics of healthy trotting horses during treadmill versus over ground locomotion. Equine Vet. J..

[18] Mendez-Angulo J.L., Firshman A.M., Groschen D.M., Kieffer P.J., Trumble T.N. (2014). Impact of walking surface on the range of motion of equine distal limb joints for rehabilitation purposes. Vet. J..

[19] Robert C., Valette J.P., Pourcelot P., AudigiÉ F., Denoix J.M. (2002). Effects of trotting speed on muscle activity and kinematics in saddlehorses. Equine Vet. J..

[20] Robert C., Valette J.P., Denoix J.M. (2000). The effects of treadmill inclination and speed on the activity of two hindlimb muscles in the trotting horse. Equine Vet. J..

[21] Crook T.C., Wilson A., Hodson-Tole E. (2010). The effect of treadmill speed and gradient on equine hindlimb muscle activity. Equine Vet. J..

[22] Tokuriki M., Ohtsuki R., KAl M., Hiraga A., Oki H., Miyahara Y., Aoki O. (1999). EMG activity of the muscles of the neck and forelimbs during different forms of locomotion. Equine Vet. J..

[23] Greco-Otto P., Bond S., Sides R., Kwong G.P., Bayly W., Léguillette R. (2017). Workload of horses on a water treadmill: Effect of speed and water height on oxygen consumption and cardiorespiratory parameters. BMC Vet. Res..

[24] Tranquille C.A., Tacey J.B., Walker V.A., Nankervis K.J., Murray R.C. (2018). International survey of equine water treadmills—Why, when, and how?. J. Equine Vet. Sci..

[25] Wilson J.M., McKenzie E., Duesterdieck-Zellmer K. (2018). International survey regarding the use of rehabilitation modalities in horses. Front. Vet. Sci..

[26] Potenza K.N., Huggons N.A., Jones A.R., Rosanowski S.M., McIlwraith C.W. (2020). Comparing racing performance following arthroscopic surgery of metacarpophalangeal/metatarsophalangeal and carpal joints in Thoroughbred racehorses rehabilitated using conventional and underwater treadmill therapies. Vet. Rec..

[27] King M.R., Haussler K.K., Kawcak C.E., McIlwraith C.W., Reiser R.F., Frisbie D.D., Werpy N.M. (2017). Biomechanical and histologic evaluation of the effects of underwater treadmill exercise on horses with experimentally induced osteoarthritis of the middle carpal joint. Am. J. Vet. Res..

[28] King M.R., Haussler K.K., Kawcak C.E., McIlwraith C.W., Reiser II R.F. (2013). Effect of underwater treadmill exercise on postural sway in horses with experimentally induced carpal joint osteoarthritis. Am. J. Vet. Res..

[29] King M., Haussler K., Kawcak C., McIlwraith C., Reiser R. (2013). Mechanisms of aquatic therapy and its potential use in managing equine osteoarthritis. Equine Vet. Educ..

[30] Greco-Otto P., Bond S., Sides R., Bayly W., Leguillette R. (2020). Conditioning equine athletes on water treadmills significantly improves peak oxygen consumption. Vet. Rec..

[31] de Meeûs d’Argenteuil C., Boshuizen B., Oosterlinck M., van de Winkel D., De Spiegelaere W., de Bruijn C.M., Goethals K., Vanderperren K., Delesalle C.J.G. (2021). Flexibility of equine bioenergetics and muscle plasticity in response to different types of training: An integrative approach, questioning existing paradigms. PLoS ONE.

[32] Van de Winkel D., de Bruijn M., Touwen N., Duchateau L., Goethals K., Oosterlinck M., Pille F., Vanderperren K., Delesalle C. Morphological changes in 15 skeletal muscles of horses after 8 weeks of aquatraining. Proceedings of the 8th International conference on Canine and Equine Locomotion (ICEL 8).

[33] Murray R.C., Hopkins E., Tracey J.B., Nankervis K., Deckers I., Mackechnie-Guire R., Tranquille C.A. Change in muscle development of horses undergoing 20 weeks of water treadmill exercise compared with control horses. Proceedings of the British Equine Veterinary Association Congress 2020: BEVA 2020.

[34] Nankervis K., Williams R. (2006). Heart rate responses during acclimation of horses to water treadmill exercise. Equine Vet. J..

[35] Wakeling J.M., Ritruechai P., Dalton S., Nankervis K. (2007). Segmental variation in the activity and function of the equine longissimus dorsi muscle during walk and trot. Equine Comp. Exerc. Physiol..

[36] Spoormakers T.J., St. George L., Smit I.H., Hobbs S.J., Brommer H., Clayton H.M., Roy S.H., Richards J., Serra Bragança F.M. (2023). Adaptations in equine axial movement and muscle activity occur during induced fore-and hindlimb lameness: A kinematic and electromyographic evaluation during in-hand trot. Equine Vet. J..

[37] St. George L.B., Spoormakers T.J., Smit I.H., Hobbs S.J., Clayton H.M., Roy S.H., Van Weeren P.R., Richards J., Serra Bragança F.M. (2022). Adaptations in equine appendicular muscle activity and movement occur during induced fore-and hindlimb lameness: An electromyographic and kinematic evaluation. Front. Vet. Sci..

[38] St. George L., Clayton H.M., Sinclair J., Richards J., Roy S.H., Hobbs S.J. (2021). Muscle Function and Kinematics during Submaximal Equine Jumping: What Can Objective Outcomes Tell Us about Athletic Performance Indicators?. Animals.

[39] Schuurman S.O., Kersten W., Weijs W.A. (2003). The Equine Hind Limb Is Actively Stabilized during Standing. J. Anat..

[40] Robert C., Valette J.P., Degueurce C., Denoix J.M. (1999). Correlation between Surface Electromyography and Kinematics of the Hindlimb of Horses at Trot on a Treadmill. Cells Tissues Organs.

[41] da Silva N.V., Bernardino Júnior R., Nomelini Q.S.S., Pereira G.F., Delfiol D.J.Z., Nogueira G.M. (2024). Electromyographic and behavioral analysis of horses submitted to medial patellar desmotomy. Vet. Res. Commun..

[42] Hermens H.J., Freriks B., Disselhorst-Klug C., Rau G. (2000). Development of Recommendations for SEMG Sensors and Sensor Placement Procedures. J. Electromyogr. Kinesiol..

[43] De Luca C.J. (1997). The use of surface electromyography in biomechanics. J. Appl. Biomech..

[44] Nankervis K., Tranquille C., McCrae P., York J., Lashley M., Baumann M., King M., Sykes E., Lambourn J., Miskimmin K.-A. (2021). Consensus for the general use of equine water treadmills for healthy horses. Animals.

[45] Roepstorff C., Dittmann M.T., Arpagaus S., Braganca F.M.S., Hardeman A., Persson-Sjödin E., Roepstorff L., Gmel A.I., Weishaupt M.A. (2021). Reliable and clinically applicable gait event classification using upper body motion in walking and trotting horses. J. Biomech..

[46] Pfau T., Witte T.H., Wilson A.M. (2005). A method for deriving displacement data during cyclical movement using an inertial sensor. J. Exp. Biol..

[47] Bragança F.S., Roepstorff C., Rhodin M., Pfau T., Van Weeren P., Roepstorff L. (2020). Quantitative lameness assessment in the horse based on upper body movement symmetry: The effect of different filtering techniques on the quantification of motion symmetry. Biomed. Signal Process. Control.

[48] Bosch S., Serra Bragança F., Marin-Perianu M., Marin-Perianu R., Van der Zwaag B.J., Voskamp J., Back W., Van Weeren R., Havinga P. (2018). EquiMoves: A wireless networked inertial measurement system for objective examination of horse gait. Sensors.

[49] Hobbs S.J., Richards J., Clayton H.M. (2014). The Effect of Centre of Mass Location on Sagittal Plane Moments around the Centre of Mass in Trotting Horses. J. Biomech..

[50] Hardeman A., Byström A., Roepstorff L., Swagemakers J., van Weeren P., Serra Bragança F. (2020). Range of motion and between-measurement variation of spinal kinematics in sound horses at trot on the straight line and on the lunge. PLoS ONE.

[51] St. George L., Hobbs S.J., Richards J., Sinclair J., Holt D., Roy S.H. (2018). The effect of cut-off frequency when high-pass filtering equine sEMG signals during locomotion. J. Electromyogr. Kinesiol..

[52] Licka T., Frey A., Peham C. (2009). Electromyographic activity of the longissimus dorsi muscles in horses when walking on a treadmill. Vet. J..

[53] Zsoldos R., Kotschwar A., Kotschwar A., Rodriguez C., Peham C., Licka T. (2010). Activity of the equine rectus abdominis and oblique external abdominal muscles measured by surface EMG during walk and trot on the treadmill. Equine Vet. J..

[54] Zsoldos R., Kotschwar A., Kotschwar A., Groesel M., Licka T., Peham C. (2010). Electromyography activity of the equine splenius muscle and neck kinematics during walk and trot on the treadmill. Equine Vet. J..

[55] Smit I.H., Hernlund E., Brommer H., van Weeren P.R., Rhodin M., Serra Braganca F.M. (2022). Continuous versus discrete data analysis for gait evaluation of horses with induced bilateral hindlimb lameness. Equine Vet. J..

[56] Hobbs S.J., Robinson M.A., Clayton H.M. (2018). A simple method of equine limb force vector analysis and its potential applications. PeerJ.

[57] Wentink G.H. (1978). Biokinetical Analysis of the Movements of the Pelvic Limb of the Horse and the Role of the Muscles in the Walk and the Trot. Anat. Embryol..

[58] Eldridge F., St George L.B., Chapman M., Harrison L., Tabor G., Uttley C., Clayton H.M. (2025). A comparison of equine hind limb muscle activation and joint motion between forward and backward walking. J. Equine Rehabil..

[59] Tokuriki M., Aoki O. (1995). Electromyographic Activity of the Hindlimb Muscles during the walk, Trot and Canter. Equine Vet. J..

[60] Zsoldos R.R., Voegele A., Krueger B., Schroeder U., Weber A., Licka T. (2018). Long term Consistency and Location Specificity of Equine Gluteus Medius Muscle Activity During Locomotion on the Treadmill. BMC Vet. Res..

[61] Prosser L.A., Stanley C.J., Norman T.L., Park H.S., Damiano D.L. (2011). Comparison of elliptical training, stationary cycling, treadmill walking and overground walking. Electromyographic patterns. Gait Posture.

[62] Lee S.J., Hidler J. (2008). Biomechanics of overground vs. treadmill walking in healthy individuals. J. Appl. Physiol..

[63] Murray M., Spurr G., Sepic S., Gardner G., Mollinger L. (1985). Treadmill vs. floor walking: Kinematics, electromyogram, and heart rate. J. Appl. Physiol..

[64] Masumoto K., Shono T., Hotta N., Fujishima K. (2008). Muscle activation, cardiorespiratory response, and rating of perceived exertion in older subjects while walking in water and on dry land. J. Electromyogr. Kinesiol..

[65] Nankervis K., Tranquille C., Tacey J., Deckers I., MacKechnie-Guire R., Walker V., Hopkins E., Newton R., Murray R. (2024). Kinematic Responses to Water Treadmill Exercise When Used Regularly within a Sport Horse Training Programme: A Longitudinal, Observational Study. Animals.

[66] von Scheven C. (2010). The Anatomy and Function of the Equine Thoracolumbar Longissimus Dorsi Muscle. Ph.D. Thesis.

[67] Tokuriki M., Otsuki R., Kai M., Hiraga A., Aoki O. (1997). Electromyographic activity of trunk muscles during locomotion on a treadmill. J. Equine Vet. Sci..

[68] Robert C., Valette J.P., Denoix J.-M. Surface electromyographic analysis of the normal horse locomotion: A preliminary report. Proceedings of the Conference on Equine Sports Medicine and Science.

[69] Robert C., Valette J., Denoix J.M. (2001). The effects of treadmill inclination and speed on the activity of three trunk muscles in the trotting horse. Equine Vet. J..

[70] Haussler K., Bertram J., Gellman K., Hermanson J. (2001). Segmental in vivo vertebral kinematics at the walk, trot and canter: A preliminary study. Equine Vet. J..

[71] Faber M., Johnston C., Schamhardt H., van Weeren R., Roepstorff L., Barneveld A. (2001). Basic three-dimensional kinematics of the vertebral column of horses trotting on a treadmill. Am. J. Vet. Res..

[72] Faber M., Schamhardt H., van Weeren R., Johnston C., Roepstorff L., Barneveld A. (2000). Basic three-dimensional kinematics of the vertebral column of horses walking on a treadmill. Am. J. Vet. Res..

