# Supplementary material for: A Feasibility Study to Determine Whether Neuromuscular Adaptations to Equine Water Treadmill Exercise Can Be Detected Using Synchronous Surface Electromyography and Kinematic Data
Source: Animals (Basel). 2025 Nov 1;15(21):3189. doi: 10.3390/ani15213189 (PMC12606775; doi:10.3390/ani15213189)
Supplement: Supplementary file 1 [file animals-15-03189-s001.zip › Supplementary Materials.pdf]

Supplementary Materials

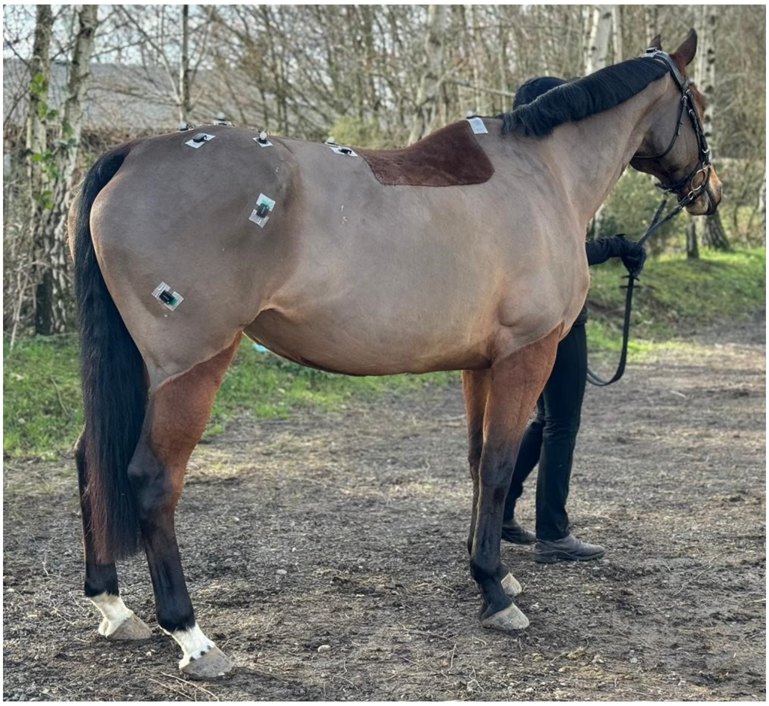

Supplementary Figure S1. Retro-reflective markers and surface electromyography (sEMG) sensors attached to the horse.

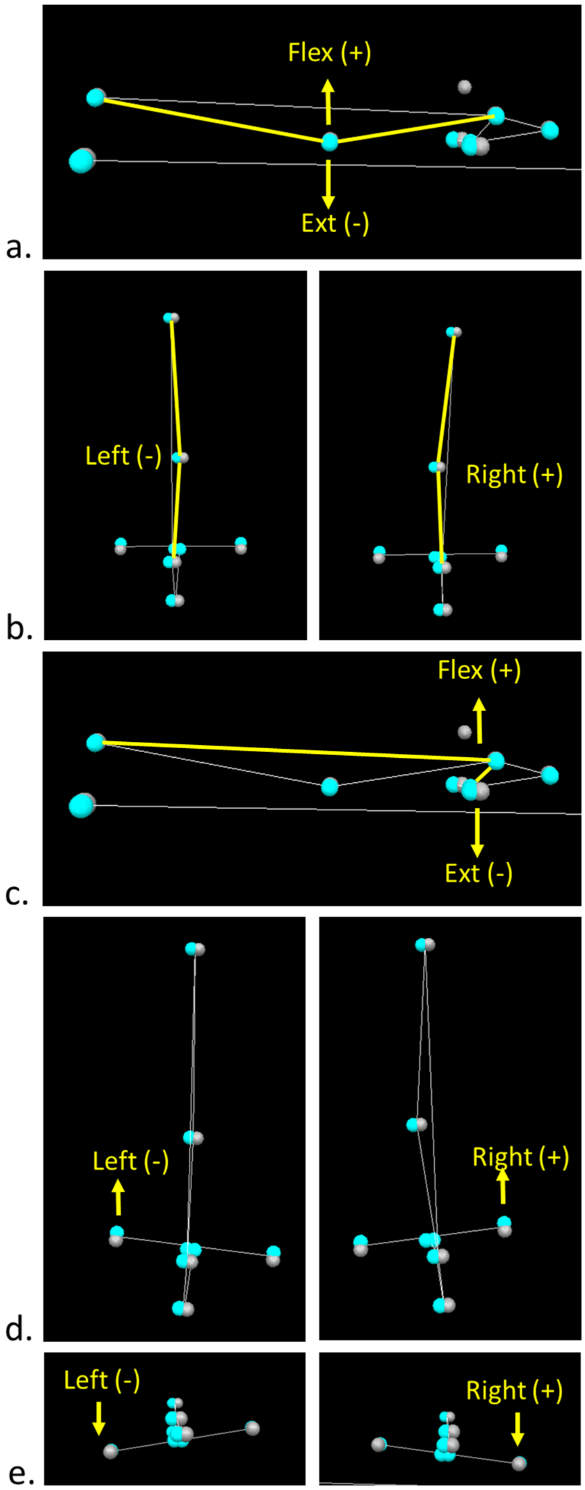

Supplementary Figure S2. Rigid-body segment model and derived kinematic angles for a) thoracolumbar flexion/extension, b) thoracolumbar lateral bending, c) pelvis pitch, d) pelvis yaw, e) pelvis roll.

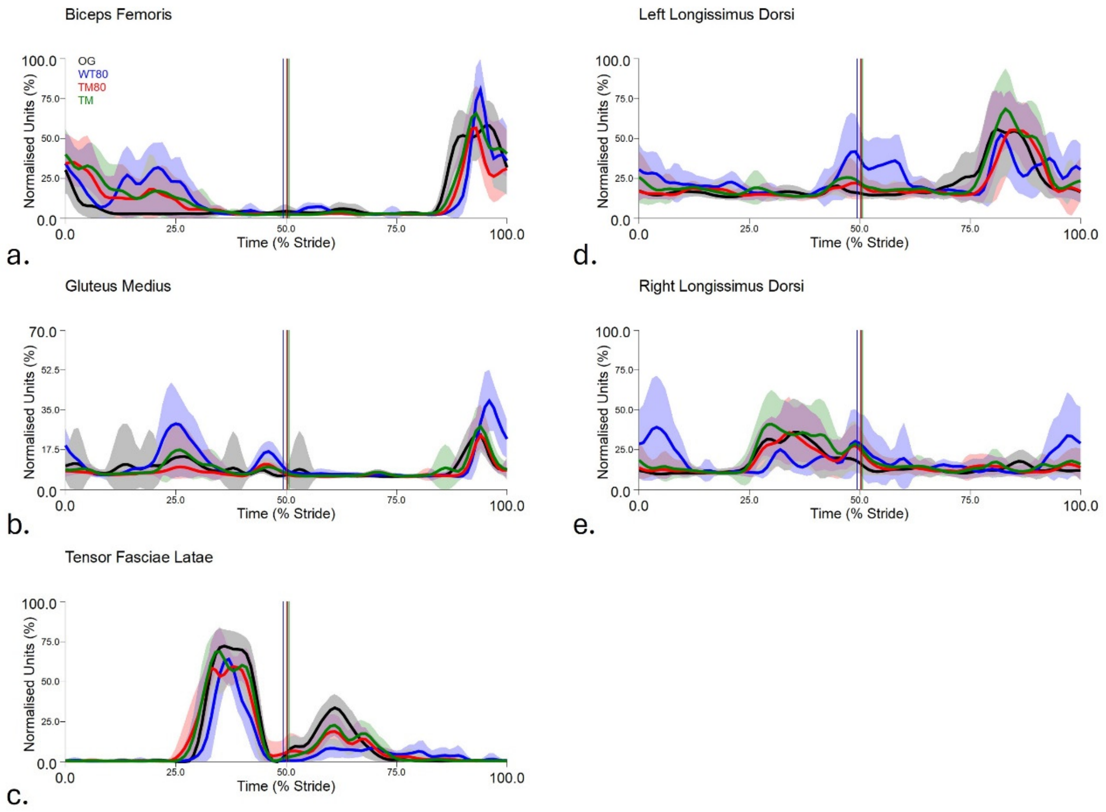

Supplementary Figure S3. Mean (solid line) and standard deviation (shaded area) time- and amplitude-normalised sEMG signals from a) biceps femoris, b) gluteus medius, c) tensor fasciae latae, d) left longissimus dorsi, e) right longissimus dorsi across OG (black), TM (green), TM_{80} (red), and WT_{80} (blue) walking conditions. sEMG signals are DC-offset, high-pass filtered (40 Hz cut-off) and smoothed using a low-pass filter (10 Hz cut-off). Vertical lines represent the average right hindlimb lift-off event for each condition. Data are time-normalized to stride duration, calculated using corresponding impacts of the right hindlimb.

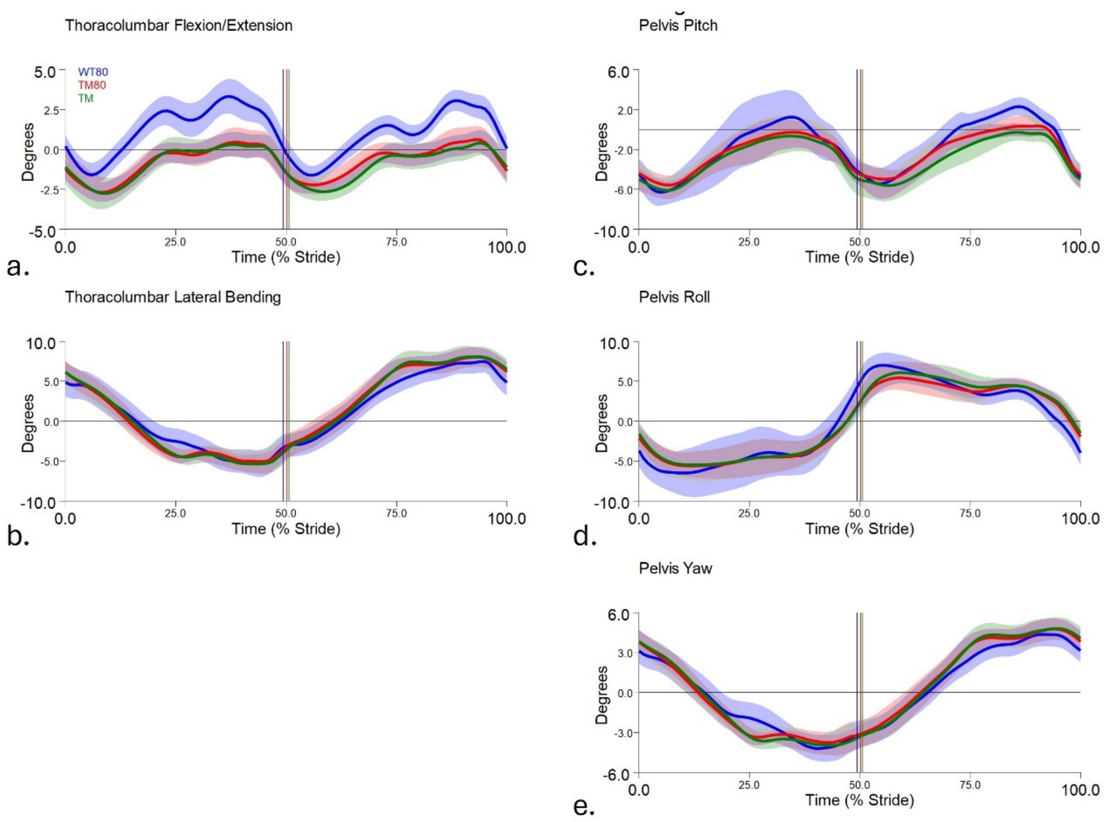

Supplementary Figure S4. Mean (solid line) and standard deviation (shaded area) time-angle curves (degrees) for a) thoracolumbar flexion/extension, b) thoracolumbar lateral bending, c) pelvic pitch, d) pelvic roll, e) pelvic yaw across TM (green), TM80 (red), and WT80 (blue) walking conditions. Signals are low-pass filtered (Butterworth 4th order, 30 Hz cut-off frequency). Vertical lines represent the average right hindlimb lift-off event for each colour-matched condition. Data are time-normalized to stride duration, calculated using corresponding impacts of the right hindlimb.

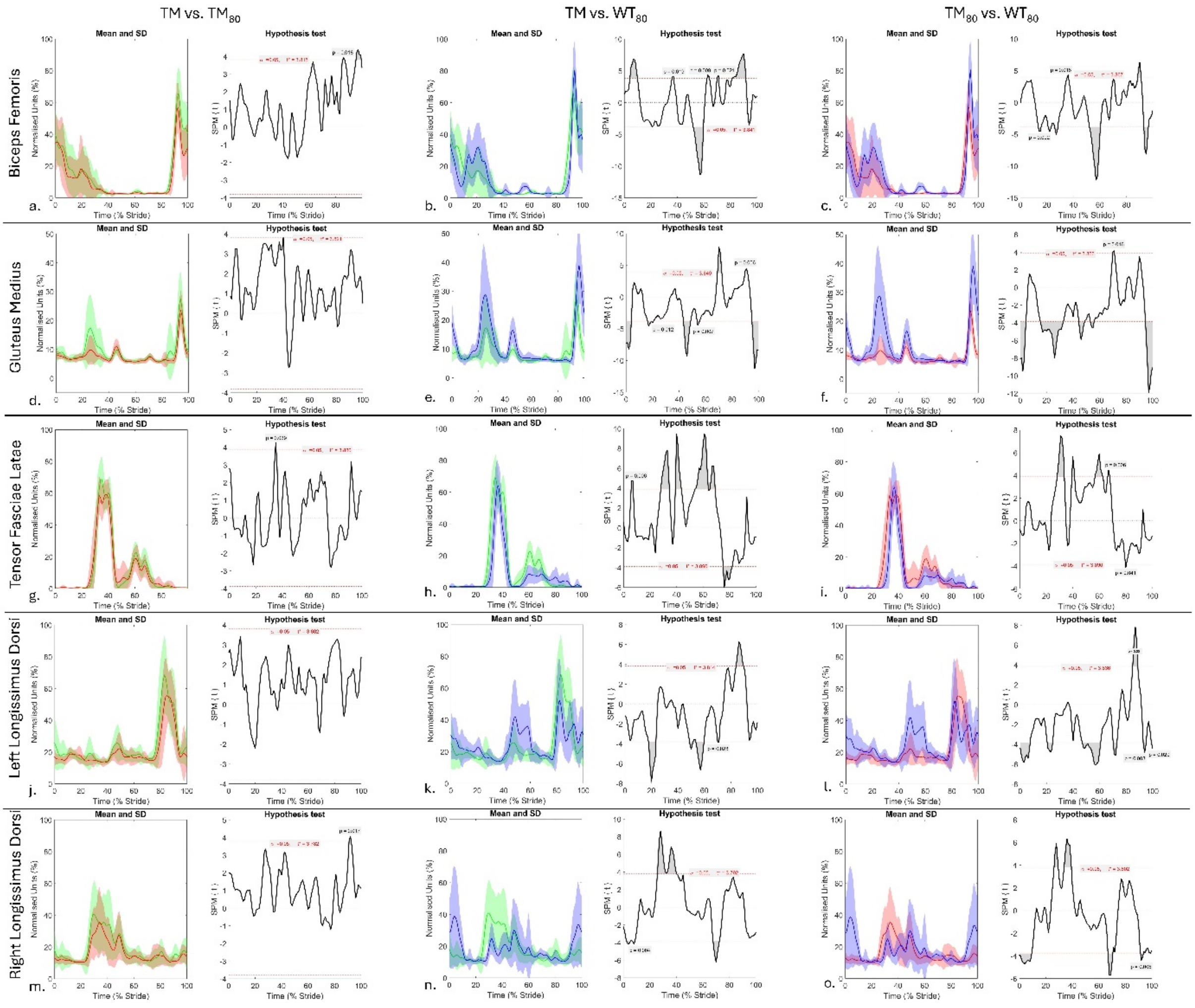

Supplementary Figure S5. SPM results for time- and amplitude- normalised sEMG data from n = 1 horse for right hindlimb muscles and bilateral longissimus dorsi between treadmill walking conditions: TM (green), TM_{80} (red), WT_{80} (blue). Within each sub-panel, left-side graphs illustrate mean (solid line) and standard deviation (shaded area) sEMG data from individual muscles that are time normalised to walk stride duration. Right-side graphs illustrate paired samples t-test SPM results (black solid line) and the critical thresholds (α, t*) for significance (red dashed line), with grey shaded areas indicating regions/data clusters with statistically significant differences ($p<0.05$) between conditions. P values are presented for data clusters with significance 0.001>0.05, all other shaded data clusters are $p<0.001$.
