# Supplementary figures and images for: A Feasibility Study to Determine Whether Neuromuscular Adaptations to Equine Water Treadmill Exercise Can Be Detected Using Synchronous Surface Electromyography and Kinematic Data
Source: Animals (Basel). 2025 Nov 1;15(21):3189. doi: 10.3390/ani15213189 (PMC12606775; doi:10.3390/ani15213189)

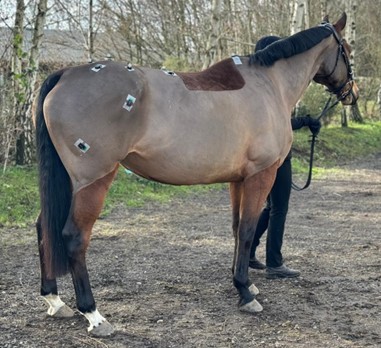

Supplement: Supplementary file 1 [file animals-15-03189-s001.zip › Supplementary Figure S1.jpg]

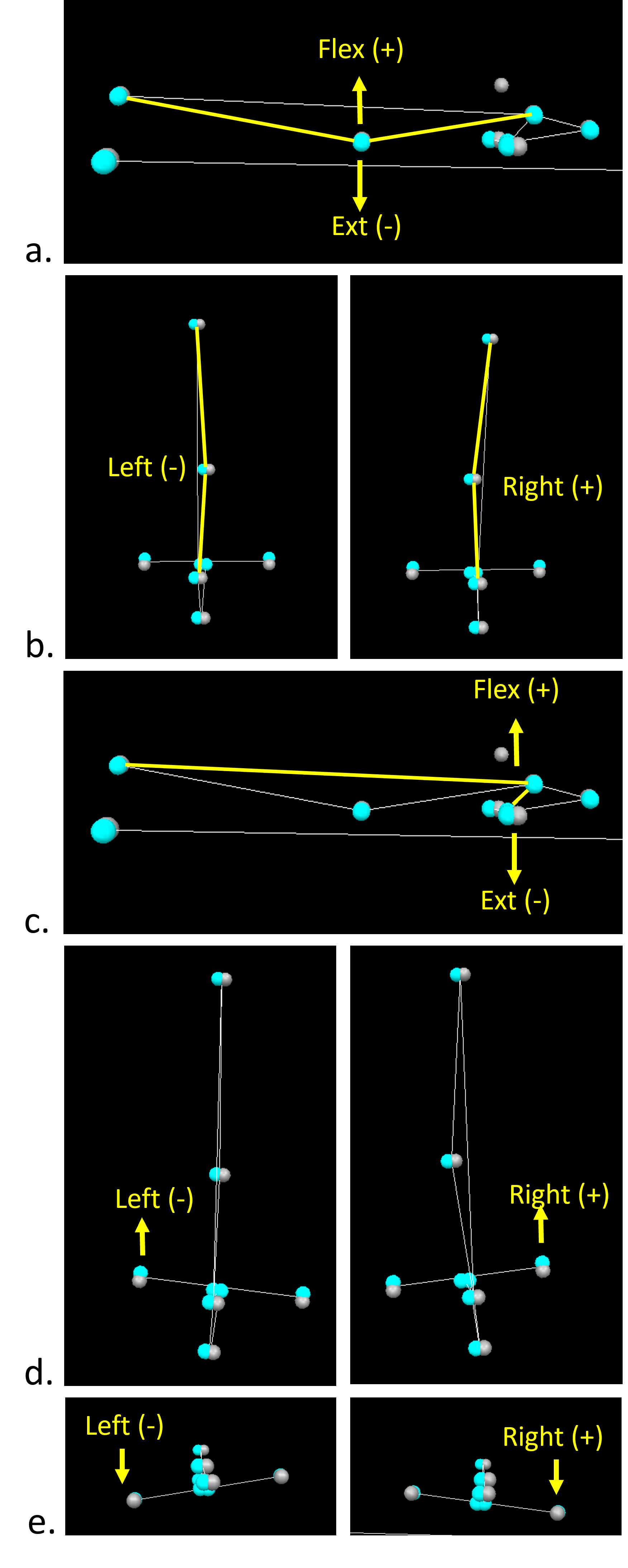

Supplement: Supplementary file 1 [file animals-15-03189-s001.zip › Supplementary Figure S2.png]

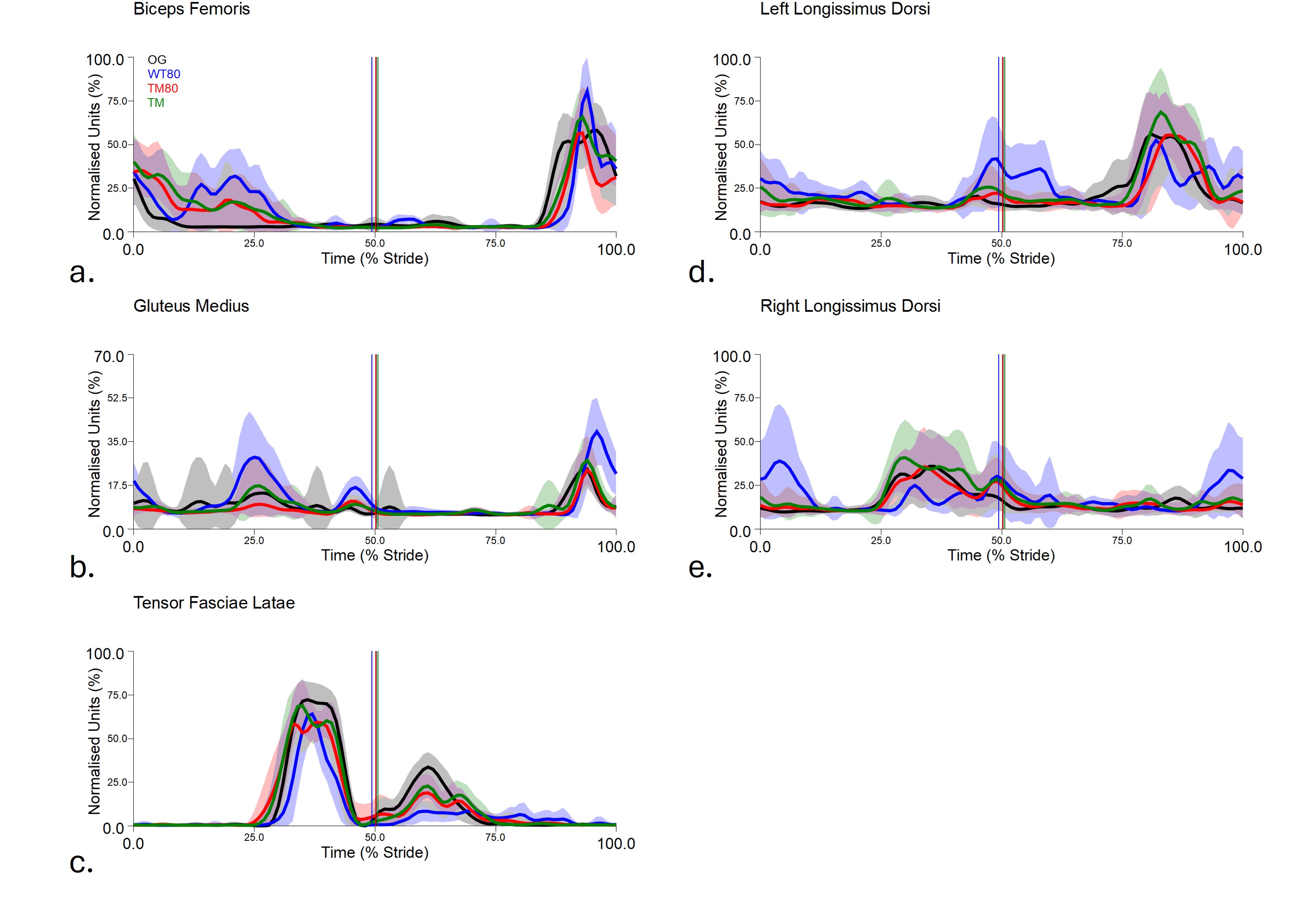

Supplement: Supplementary file 1 [file animals-15-03189-s001.zip › Supplementary Figure S3.jpg]

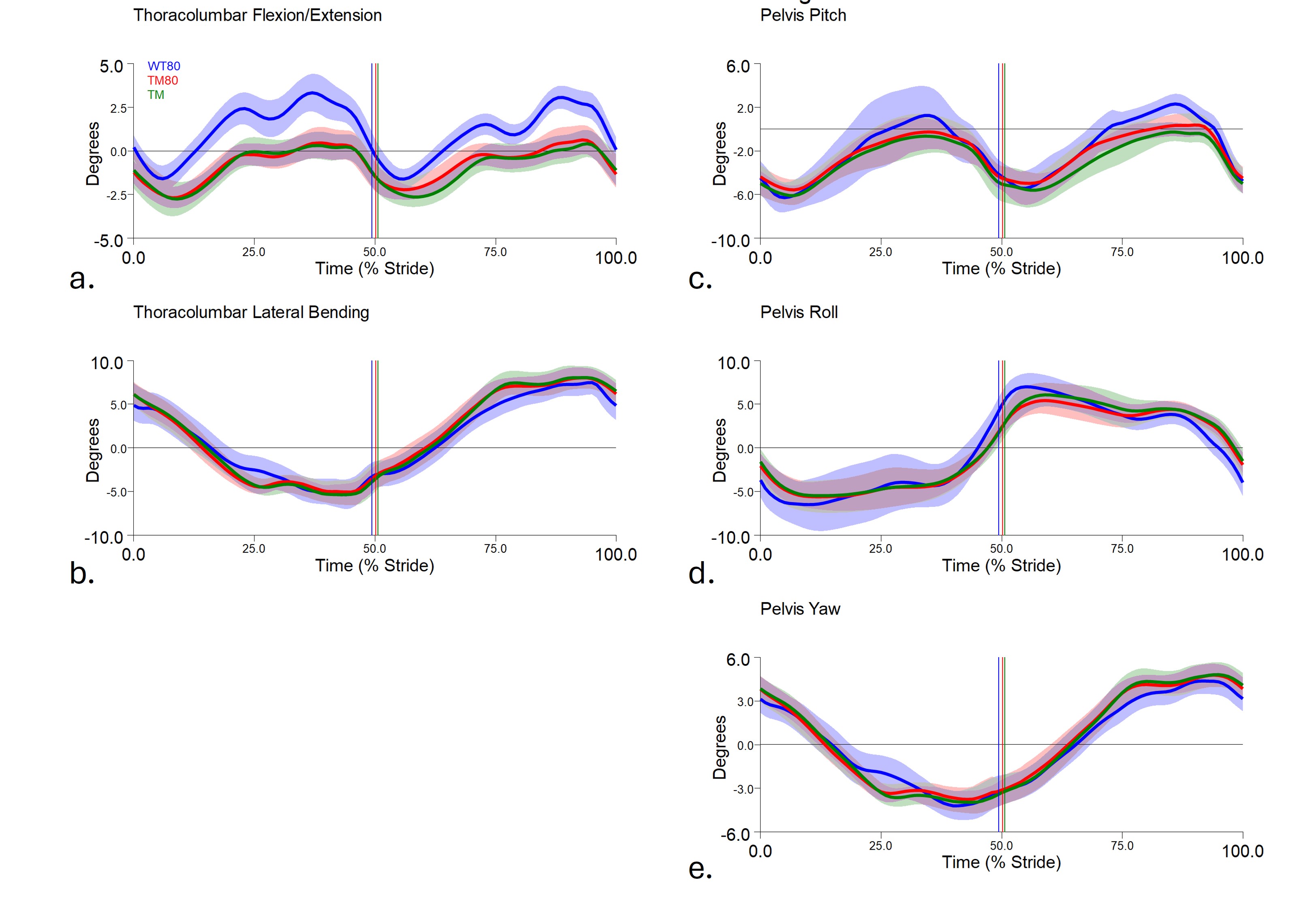

Supplement: Supplementary file 1 [file animals-15-03189-s001.zip › Supplementary Figure S4.jpg]
